# The Zinc-Finger Domain Containing Protein ZC4H2 Interacts with TRPV4, Enhancing Channel Activity and Turnover at the Plasma Membrane

**DOI:** 10.3390/ijms21103556

**Published:** 2020-05-18

**Authors:** Laura Vangeel, Annelies Janssens, Irma Lemmens, Sam Lievens, Jan Tavernier, Thomas Voets

**Affiliations:** 1 Laboratory of Ion Channel Research, VIB-KU Leuven Center for Brain & Disease Research, 3000 Leuven, Belgium; laura.vangeel@kuleuven.vib.be (L.V.); annelies.janssens@kuleuven.vib.be (A.J.); 2 Department of Cellular and Molecular medicine, KU, 3000 Leuven, Belgium; 3 Cytokine Receptor Laboratory, Faculty of Medicine and Health Sciences, Department of Biomolecular Medicine, Ghent University, and Center for Medical Biotechnology, VIB, 9052 Ghent, Belgium; irma.lemmens@vib-ugent.be (I.L.); slievens@orionisbio.com (S.L.); jan.tavernier@vib-ugent.be (J.T.)

**Keywords:** TRPV4, protein–protein interaction, ZC4H2, Ca^2+^ signaling, channel turnover

## Abstract

The Ca^2+^-permeable Transient Receptor Potential channel vanilloid subfamily member 4 (TRPV4) is involved in a broad range of physiological processes, including the regulation of systemic osmotic pressure, bone resorption, vascular tone, and bladder function. Mutations in the *TRPV4* gene are the cause of a spectrum of inherited diseases (or TRPV4-pathies), which include skeletal dysplasias, arthropathies, and neuropathies. There is little understanding of the pathophysiological mechanisms underlying these variable disease phenotypes, but it has been hypothesized that disease-causing mutations affect interaction with regulatory proteins. Here, we performed a mammalian protein–protein interaction trap (MAPPIT) screen to identify proteins that interact with the cytosolic N terminus of human TRPV4, a region containing the majority of disease-causing mutations. We discovered the zinc-finger domain-containing protein ZC4H2 as a TRPV4-interacting protein. In heterologous expression experiments, we found that ZC4H2 increases both the basal activity of human TRPV4 as well as Ca^2+^ responses evoked by ligands or hypotonic cell swelling. Using total internal reflection fluorescence (TIRF) microscopy, we further showed that ZC4H2 accelerates TRPV4 turnover at the plasma membrane. Overall, these data demonstrate that ZC4H2 is a positive modulator of TRPV4, and suggest a link between TRPV4 and ZC4H2-associated rare disorders, which have several neuromuscular symptoms in common with TRPV4-pathies.

## 1. Introduction

The transient receptor potential (TRP) superfamily consists of polyvalent ion channels expressed throughout the whole body. The 27 mammalian members, which are subdivided into six families (A: ankyrin, C: canonical, M: melastatin, ML: mucolipin, P: polycystin, V: vanilloid) based on amino acid homology, play important roles in cellular signaling and a variety of physiological processes, including mineral and glucose homeostasis, cardiac rhythmicity, kidney function, taste, and somatosensation. In line with their important physiological roles, mutations in several TRP channel genes are the cause of monogenic human diseases [1].

One particular member, TRPV4, shows a complex relationship between gene mutation and disease. TRPV4 is a mechanosensitive channel expressed in multiple tissues, including the brain, bone, and various epithelial/endothelial cell layers [2]. Moderate heat, cell swelling, and several endogenous (e.g., arachidonic acid and 5’,6’-epoxyeicosatrienoic acid) and synthetic (e.g., 4 alpha-phorbol 12,13-didecanoate (4α-PDD), GSK1016790A) ligands activate this non-selective cation channel. The broad expression and polymodal gating of TRPV4 is reflected in a wide range of physiological functions, and in the complex phenotype of TRPV4 knockout in mice. Although viable and fertile, TRPV4 knockout mice suffer from compromised vascular endothelial function, deficits in osmosensation, a higher bone density, and alterations in bladder function [2]. Mutations in the human *TRPV4* gene give rise to a broad spectrum of disease phenotypes, known as TRPV4-pathies [3,4,5,6,7,8]. At this point, more than 70 mutations in the *TRPV4* gene have been identified [2], and the resulting TRPV4-pathy disease spectrum can be classified in three different groups based on the key symptoms [6]. A first group includes the skeletal dysplasias (SDs), which are characterized by abnormalities of bone and cartilage growth, malformations in the skeleton, platyspondyly, and defects in bone ossification [7,9,10]. A second group are the neuropathies, including distal or scapuloperoneal spinal muscular atrophy (SMA) and Charcot–Marie–Tooth (CMT) type 2C, which mainly present with motor and sometimes sensory defects [3,4,5,6]. Thirdly, TRPV4 mutations are the cause of a rare and aggressive osteoarthropathy of the fingers and toes, known as digital arthropathy-brachydactyly (FDAB) [11]. Note, however, that in clinical practice the classification is often less strict, and patients sometimes exhibit a variable mixture of skeletal, motor, and neuronal symptoms [6,12].

It is intriguing to note that some mutations affect the bones and joints without signs of neuropathy, others lead to neuropathies without obvious skeletal pathology, and still others lead to a mixed phenotype. At this moment, there is no clear understanding of the link between specific mutations and the resulting pathology [6,8]. Although there seems to be some mutation hotspots, in particular the N-terminal Ankyrin repeat domain (ARD) or in the transmembrane segments, the location or nature of the mutations do not allow prediction of the actual disease phenotype. Most strikingly, mutation of the residue at position 183 of TRPV4 can cause a skeletal dysplasia [10] or CMT type 2C [13] depending on the introduced amino acid, with no phenotypic overlap between the cases. Moreover, there are substantial interfamilial differences regarding onset, symptoms, and severity of the disease. In some cases, TRPV4-pathies are accompanied with further symptoms, such as hearing loss, disturbed temperature regulation, and vocal cord paralysis [12,14].

It has been suggested that the divergent pathological effects of the disease-causing TRPV4 mutations in various tissues may involve altered interaction with cell-specific regulatory proteins [6]. In this study, we made use of the mammalian protein–protein interaction trap (MAPPIT) [15] to search for proteins that interact with the cytosolic N-terminus of TRPV4. We identified the zinc-finger C4H2-type containing protein (ZC4H2) as a TRPV4 interaction partner, and provide evidence that it augments TRPV4-mediated Ca^2+^ signals and enhances channel turnover at the plasma membrane. Interestingly, mutations in the X-linked *ZC4H2* gene are the cause of so-called ZC4H2-associated rare disorders (ZARDs) [16]), formerly referred to as Wieacker–Wolff syndrome or Miles–Carpenter syndrome. ZARD patients suffer from a complex set of neurological problems, including several symptoms that overlap with typical features of TRPV4-pathies, such as arthrogryposis, distal muscle weakness, club foot, and camptodactyly. Our results thus describe a newly identified TRPV4 interactor, and suggest a potential link between ZARD and TRPV4-pathies.

## 2. Results

### 2.1. Identification of ZC4H2 as a Novel TRPV4 Interactor

We hypothesized that cell-specific regulatory proteins binding to and shaping TRPV4 function could play a role in the pathophysiology of TRPV4-pathies. In order to discover proteins interacting with human TRPV4, we performed a MAPPIT screening [15]. MAPPIT is a two-hybrid technology based on the functional complementation of a human type 1 cytokine receptor. It is only when bait and prey physically interact that a functional receptor is obtained. Ligand binding then leads to cross activation of associated Janus kinases, which in turn phosphorylate and activate signal transducer and activator of transcription (STAT) complexes. After migration to the nucleus, these complexes induce specific target gene transcription. In this experimental set-up, STAT complexes trigger the expression of a bioluminescence enzyme luciferase. This simple read-out enables high-throughput screening in a human cellular context, for covalent as well as transient and indirect interactions.

**Figure 1:**
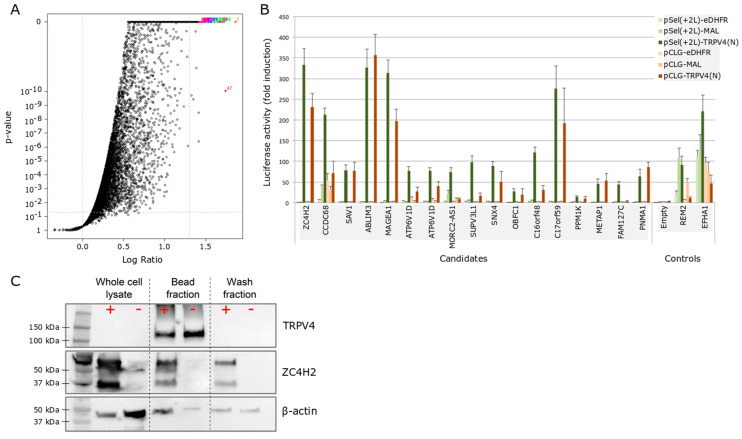
MAPPIT and co-IP show an interaction of ZC4H2 and TRPV4. (**A**) Volcano plot of the whole protein library tested in an initial MAPPIT screen, with the *p*-value in the function of the MAPPIT signal. Interesting ‘hits’ are located at the upper right corner. (**B**) Luciferase read-out of the top 17 hits identified in the MAPPIT screening. Baits cloned in the pSEL and pCLG vector background were tested in parallel and contained either the human N-terminus TRPV4 or an irrelevant protein (E. coli dihydrofolate reductase (eDHFR) or myelin and lymphocyte protein (MAL)). Additionally, an empty prey plasmid was used as a negative control. Positive assay controls are RRAD and GEM Like GTPase 2 (REM2) and EF-hand domain family member A1 (EFHA1), two proteins known to bind the cytokine receptor complex bait independently. (**C**) Human embryonic kidney (HEK)-293T cells were co-transfected with TRPV4-GFP and ZC4H2-mCherry (+) or TRPV4-GFP and mCherry (−). SDS-PAGE was performed for three conditions: the whole cell lysate, the fraction bound to GFP-Trap^®^ beads, and the unbound (wash) fraction. Staining was done using specific antibodies for human TRPV4 (98 kDa), ZC4H2 (26 kDa), and β-actin (42 kDa). Note that in these experiments, TRPV4 and ZC4H2 are coupled to green fluorescent protein (GFP) and mCherry, respectively, which increases the molecular weights of the detected proteins by 27 kDa. In two independent experiments, we confirmed that there was negligible binding of mCherry and ZCH2-mCherry to the GFP-Trap^®^ beads.

Considering the mutational hot spot in the ARD, a protein domain susceptible for protein interaction [17], we used the N-terminus of TRPV4 (AA 1–466) as bait. As possible preys, the human ORFeome v8.1 and ORFeome Collaboration (OC) collection was used, containing 14,817 clones [18,19]. After a primary screening of the full prey collection (Figure 1A), 17 top hits were retested in a double plasmid system (TRPV4 N-terminus in pSEL and pCLG-plasmid backbone) with multiple controls, to assure signal specificity (Figure 1B). Based on the amplitude and specificity of the luciferase signals (Figure 1B), as well as on a literature search for known functions and subcellular localization of confirmed hits, we initially focused on three cytosolic proteins: ZC4H2, abLIM3, and PNMA1.

**Figure A1:**
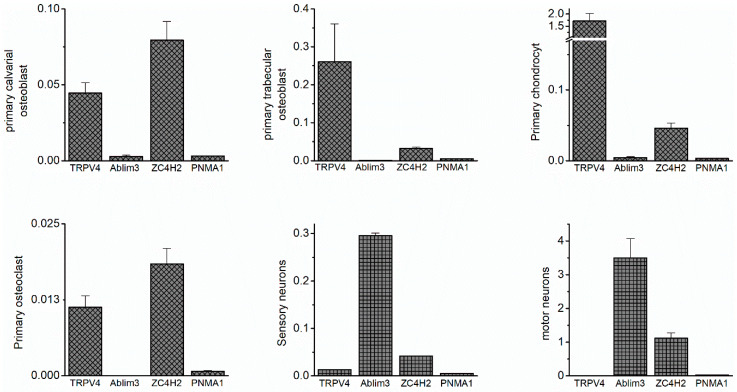
Initial evaluation of other MAPPIT hits. mRNA expression of TRPV4 and the indicated MAPPIT hits in the indicated mouse tissues. Values were normalized to the internal control HPRT. PNMA1 expression was low in all samples, while abLIM3 was primarily found in tissues with low TRPV4 expression.

For these candidates, we performed qPCR experiments to evaluate their expression in cell types relevant for TRPV4-pathies (Figure A1), including osteoclasts, osteoblasts, chondrocytes, sensory neurons, and motor neurons. Levels of PNMA1 (paraneoplastic Ma antigen 1) were low or undetectable in all tested cell types. In osteoclasts, osteoblasts, and chondrocytes, where TRPV4 expression was the highest (Figure A1), mRNA levels of actin binding LIM protein 3 (abLIM3) were low or below the detection level, while higher levels were found in sensory neurons and motor neurons, where TRPV4 expression is low. Finally, the qPCR experiments showed substantial co-expression of TRPV4 and ZC4H2 in osteoblasts, osteoclasts, and chondrocytes. 

Interestingly, patients with mutations in the X-linked gene encoding the zinc-finger domain-containing protein ZC4H2 exhibit a highly variable clinical presentation, originally described as Wieacker–Wolff or Miles–Carpenter syndrome but recently confined into ZC4H2 deficiency or ZC4H2-associated rare disorders (ZARDs) [16]. Generally, patients are diagnosed with intellectual disability accompanied by variable symptoms of central and peripheral nervous system involvement, including spasticity, hyperreflexia, muscle weakness, and arthrogryposis [16,20], symptoms that are also present to a variable extent in patients with mutations in the *TRPV4* gene [14]. However, at this point, very little is known about the molecular expression, cellular function, and (patho)physiological roles of ZC4H2. The 26-kD protein, encoded on the X chromosome, contains a C-terminal zinc-finger domain, a coiled-coil region, and a nuclear localization signal. At the subcellular level, ZC4H2 partitions between the nucleus and cytoplasm [20]. Expression of ZC4H2 is predominantly found in the brain and central nervous system (CNS) during embryonic development, where it is involved in neuronal development [20,21], possibly via interaction with binding partners, such as Small Mothers Against Decapentaplegic homolog 3 (Smad3) and Ring Finger Protein 220 (RNF220) [22,23]. However, a clear molecular mechanism of ZC4H2 function or dysfunction has not yet been described. Considering the partial overlap of symptoms between ZARDs and TRPV4-pathies, and the substantial co-expression in relevant cell types, we focused our research on a potential functional interaction between ZC4H2 and TRPV4.

### 2.2. ZC4H2 Binds to TRPV4 and Enhances Channel Activity

As a first step, we confirmed the results from the MAPPIT screen by showing co-immunoprecipitation of TRPV4-GFP and ZC4H2-mCherry when co-expressed in HEK-293T cells (Figure 1C). 

**Figure 2:**
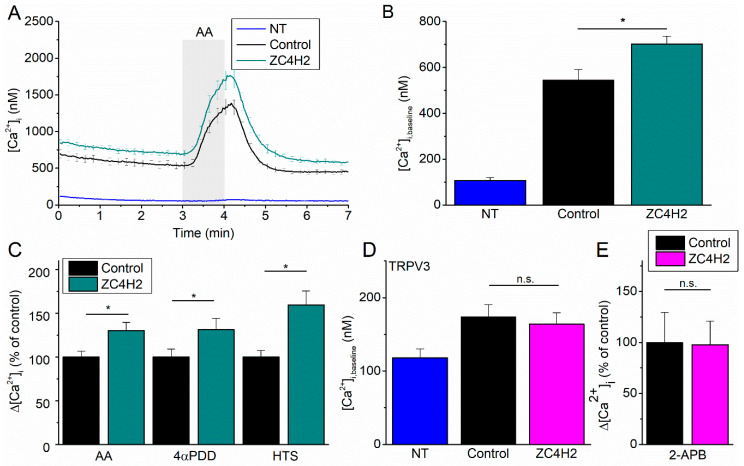
Effect of ZC4H2 on TRPV4 channel activity. (**A**) Time course of the intracellular Ca^2+^ concentration ([Ca^2+^]_i_) (mean ± SEM) in non-transfected (NT) HEK-293T cells (*n* = 30), and cells co-transfected with TRPV4 and mCherry (control, *n* = 79) or ZC4H2-mCherry (*n* = 80), upon stimulation with arachidonic acid (AA; 10 µM). (**B**) Mean baseline [Ca^2+^]_i_ in experiments as in (A). (**C**) Normalized [Ca^2+^]_i_ amplitudes in response to the TRPV4-activating stimuli AA (10 µM), 4α-PDD (10mM), or hypotonic solution. Values are normalized to the response of cells expressing TRPV4 and mCherry. Representative traces are shown in Figure A3. (**D**) Co-expression of ZC4H2 is without effect on baseline [Ca^2+^]_i_ in cells expressing TRPV3 (*n* = 144 for ZC4H2 co-transfected, 183 for control, *n* = 26 for non-transfected). (**E**) Normalized [Ca^2+^]_i_ amplitudes in TRPV3-expressing cells in response to the agonist 2-APB (25 µM). Values are normalized to the response of cells expressing TRPV3 and mCherry. *p*-values < 0.05 were considered significant (*).

**Figure A2:**
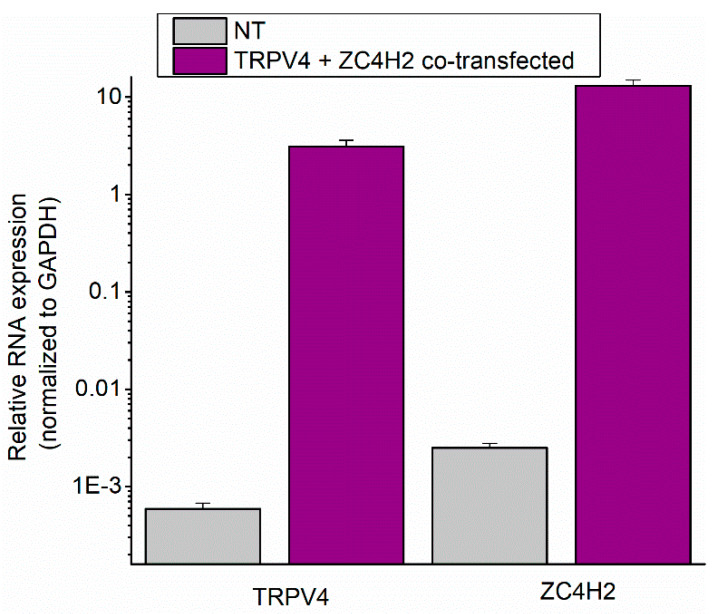
Expression levels of TRPV4 and ZC4H2 in HEK-293T cells. mRNA expression of TRPV4 and ZC4H2 in non-transfected (NT) cells and cells co-transfected with TRPV4-GFP and ZC4H2-mCherry.

**Figure A3:**
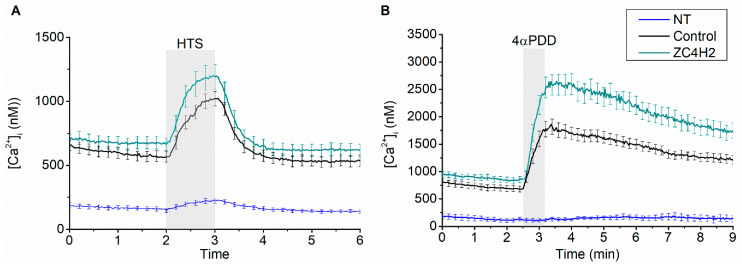
Effect of ZC4H2 on TRPV4 function is not stimulus specific. Representative time course traces of the intracellular Ca^2+^ concentration ([Ca^2+^]_i_) (mean ± SEM) in non-transfected (NT) HEK-293T cells (*n* = 30), and cells co-transfected with TRPV4 and mCherry or TRPV4 and ZC4H2-mCherry, upon stimulation with (**A**) HTS and (**B**) 4αPDD (10µM).

Next, we evaluated whether ZC4H2 influences TRPV4 function. We performed Fura-2-based imaging of the intracellular calcium concentration ([Ca^2+^]_i_) in HEK-293T cells expressing human TRPV4, which were co-transfected with either human ZC4H2 coupled to mCherry or with mCherry as the control. At the mRNA level, expression of TRPV4 and ZC4H2 was very low in non-transfected HEK-293T, and increased by more than three orders of magnitude upon transfection (Figure A2). We observed a significant increase in baseline [Ca^2+^]_i_ levels in the cells co-expressing ZC4H2 compared to the control (Figure 2A,B), indicating that basal TRPV4 activity is enhanced. Furthermore, the amplitude of the [Ca^2+^]_i_ rise in response to TRPV4 agonists was significantly larger in the presence of ZC4H2 (Figure 2A,C). This effect was stimulus independent, as it was observed with different modes of activation, including the synthetic chemical agonists 4α-PDD, the endogenous ligand arachidonic acid, and hypotonic cell swelling (Figure 2C, Figure A3). The decay of the [Ca^2+^]_i_ signal following TRPV4 activation by AA could be well fitted by a monoexponential function, and the resulting time constants were similar with or without ZC4H2 (243 ± 16 s for ZC4H2 versus 235 ± 7 s for control), which suggests that intracellular Ca^2+^ extrusion mechanisms were not markedly affected. To investigate the specificity of the effect of ZC4H2, we performed similar experiments on TRPV3, the closest homologue of TRPV4. ZC4H2 had no effect on baseline [Ca^2+^]_i_ or on the response to the ligand 2-Aminoethoxydiphenyl borate (2-APB) (Figure 2D,E). These results indicate that ZC4H2 does not affect the functional expression of TRPV3, and also argue against a general effect of ZC4H2 on intracellular Ca^2+^ handling. 

### 2.3. TIR-FRAP Experiments Unravel the Effect on TRPV4 Turnover at the Plasma Membrane

**Figure 3:**
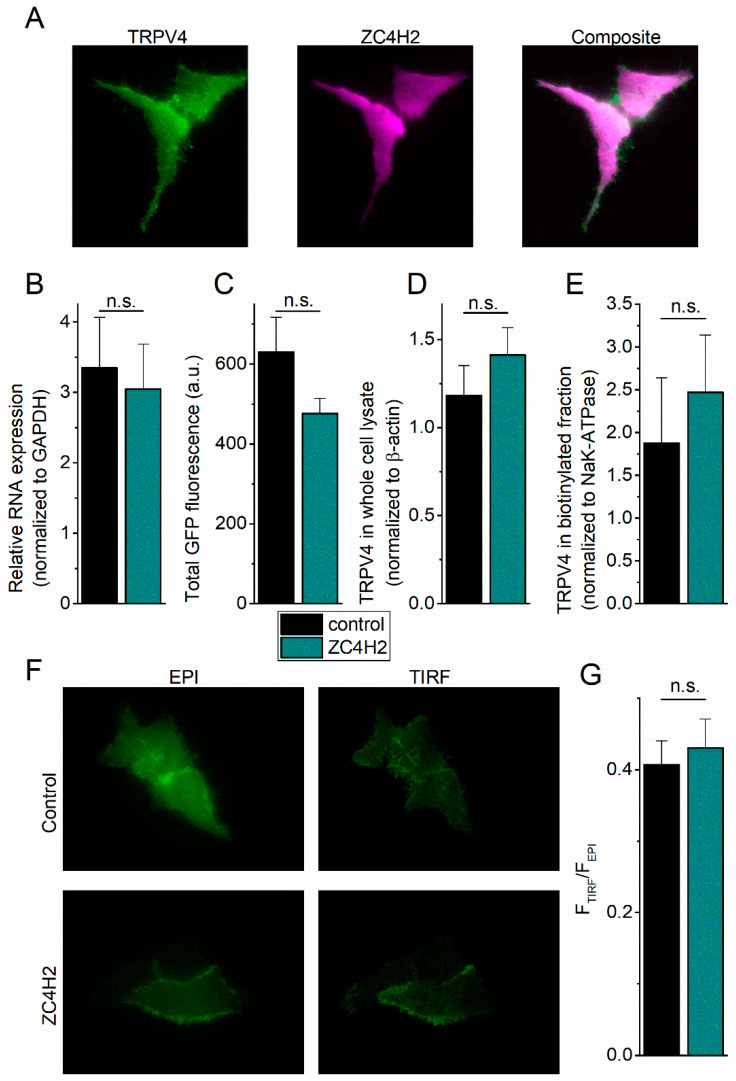
ZC4H2 does not affect the expression or subcellular localization of TRPV4. (**A**) Representative TIRF images showing the localization of heterologously expressed TRPV4-GFP and ZC4H2-mCherry. (**B**) Relative mRNA expression of TRPV4 in cells co-transfected with ZC4H2 or mCherry control. Data are normalized to the housekeeping gene Glyceraldehyde-3-Phosphate Dehydrogenase (GAPDH). (*n* = 11) (**C**) Mean TRPV4-GFP fluorescence (EPImode). (*n* = 245 for ZC4H2, 220 for control) (**D**) Mean TRPV4 expression in whole cell lysate after SDS-PAGE. (*n* = 14) (**E**) Mean TRPV4 expression in the biotinylated fraction, normalized to the NaK-ATPase (plasma membrane marker). (*n* = 8) (**F**) Representative images showing TRPV4 GFP fluorescence measured in epifluorescence (EPI) mode and TIRF mode in cells co-transfected with ZC4H2 or mCherry control. (**G**) Ratio of TRPV4-GFP fluorescence in TIRF mode versus EPI mode, as an estimate of the distribution of TRPV4-GFP between the bulk cell and the perimembrane area in close vicinity of the coverslip. (*n* = 24 for ZC4H2, *n* = 21 control). Values are mean ± SEM.

**Figure A4:**
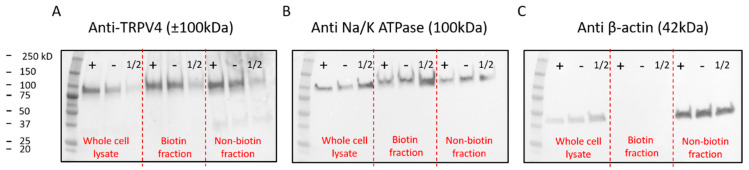
Biotinylation experiments did not reveal differences in TRPV4 membrane expression. Example blot probed for (**A**) TRPV4, (**B**) the membrane marker Na/K ATPase, and (**C**) β-actin. The protein ladder (left) is followed by three lanes of whole-cell lysis samples, middle three lanes demonstrate the biotinylated membrane fraction, while the last three lanes consist of the non-biotinylated fraction. Samples are, from left to right; (‘+’) HEK-293T cells co-transfected with TRPV4 and ZC4H2, (‘−’) HEK-293T cells co-transfected with TRPV4 and control, (‘1/2’) HEK-293T cells transfected with a half concentration of TRPV4 (0.5 µM).

When visualized using TIRF imaging, TRPV4 exhibited a typical plasma membrane staining, including expression in fine membrane protrusions, whereas the cytosolic protein ZC4H2 showed a uniform distribution in the cell, including but not limited to areas with high TRPV4 expression (Figure 3A). Consistent with the fact that ZC4H2 is a cytosolic protein and with the observation that only a fraction of total ZC4H2 is bound to TRPV4 (Figure 1C), we did not observe specific colocalization of both proteins. There are several potential mechanisms that may explain the increased basal [Ca^2+^]_i_ and TRPV4-dependent stimulus responses in the presence of ZC4H2, including higher levels of channel expression, increased channel activation, or enhanced channel transport to the plasma membrane. First, we performed qPCR to address whether co-expression of ZC4H2 affects TRPV4 expression at the mRNA level. This analysis did not reveal any difference in the mRNA levels between cells expressing ZC4H2 or the mCherry control (Figure 3B). Secondly, both the mean cellular GFP fluorescence (Figure 3C) as well as the total TRPV4 protein expression in whole cell lysates (Figure 3D, Figure A4) were similar in the control and ZC4H2-expressing cells, indicating that ZC4H2 does not affect TRPV4 expression at the protein level. Third, biotinylation experiments to specifically probe for plasma membrane proteins did not reveal an effect of ZC4H2 on TRPV4 levels in the plasma membrane (Figure 3E, Figure A4). To further test this conclusion, we performed combined epifluorescence and TIRF imaging experiments to compare the distribution of TRPV4 between the perimembrane area and the bulk cytosol. The ratio of GFP fluorescence in the TIRF mode versus epifluorescence mode was unchanged in the absence or presence of ZC4H2 (Figure 3F,G). Taken together, these data indicate that ZC4H2 does not increase the expression levels of TRPV4 nor its steady-state presence at the plasma membrane. 

**Figure 4:**
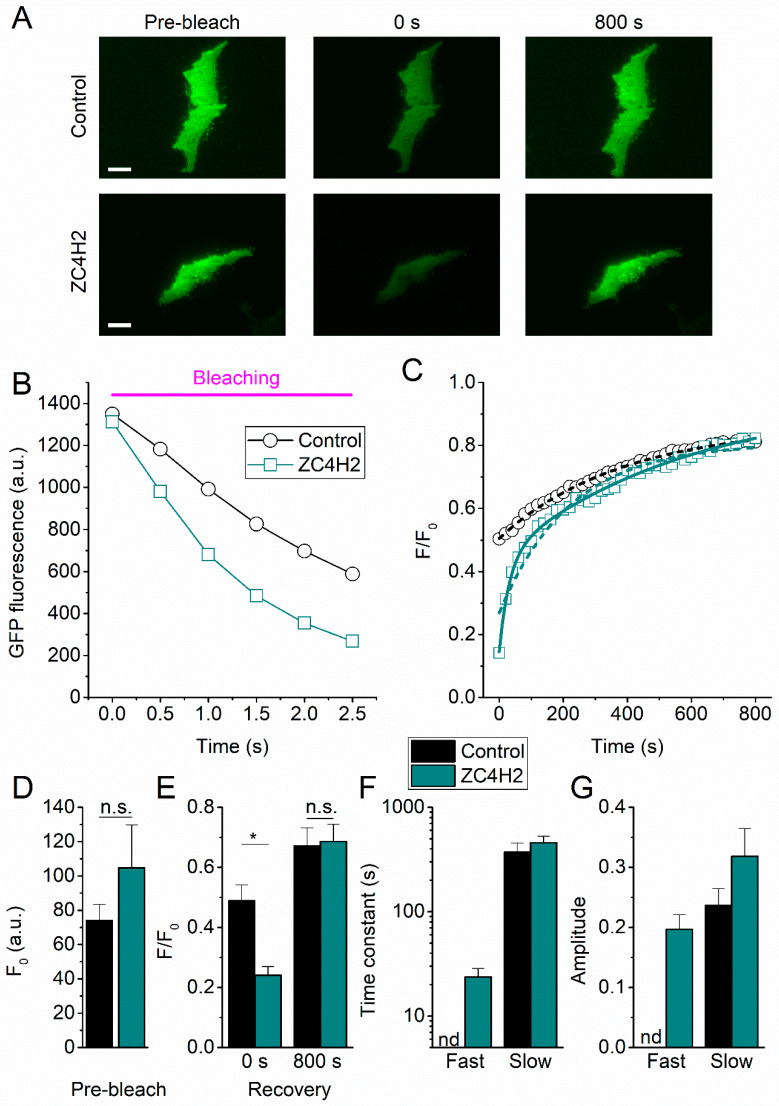
Effect of ZC4H2 on TRPV4 turnover at the plasma membrane assayed using TIR-FRAP. (**A**) TIRF images of TRPV4-GFP in HEK-293T cells co-expressing mCherry (control) or ZC4H2-mCherry. Images were taken before bleaching, and at 0 and 800s after bleaching. Scale bar = 10 µm. (**B**,**C**) Time course of the decay of TRPV4-GFP fluorescence during the bleaching process (**B**) and the recovery of the fluorescence following bleaching (**C**) in representative cells expressing ZC4H2 or the control. Dotted and solid lines in C represent mono- and bi-exponential fits, respectively. (**D**) Mean basal TRPV4-GFP fluorescence before bleaching, in control and ZC4H2-expressing cells. (**E**) Mean TRPV4-GFP fluorescence at 0 and 800s after bleaching. (**F**,**G**) Time constants and corresponding relative amplitudes obtained from exponential fits to recovery time courses as in (**C**). In control cells, a mono-exponential fit was generally sufficient to describe the recovery process. In ZC4H2-expressing cells, adequate fitting required a second faster kinetic component. Number of cells in (**D**–**G**): control: *n* = 16; ZC4H2: *n* = 14. *p*-values < 0.05 were considered significant (*).

Dynamic changes in TRP channel availability at the membrane may constitute an important regulatory mechanism for TRP channel function in vivo [24]. Yet, detailed knowledge regarding cellular trafficking kinetics or mechanisms of membrane incorporation and retrieval is available for only a limited number of TRP channels [25,26]. To characterize the perimembrane dynamics of TRPV4 in the absence or presence of ZC4H2, we performed total internal reflection fluorescence recovery after photo-bleaching (FRAP) experiments (Figure 4A). In these experiments, we used a TIRF laser to selectively bleach the fluorescently labeled TRPV4 in close proximity of the glass–plasma membrane interface (Figure 4B), and followed the recovery of the GFP fluorescence in the evanescent field as a measure of the transport of new unbleached TRPV4-GFP from the cell interior to the plasma membrane (Figure 4C). Intriguingly, whereas the pre-bleaching (baseline) TRPV4-GFP fluorescence was not different between control and ZC4H2-expressing cells, (Figure 4D), we observed significantly more extensive bleaching in the presence of ZC4H2 (Figure 4E). Following bleaching, fluorescence recovery was also clearly enhanced in the ZC4H2-expressing cells, such that the final recovered fluorescence at the end of the experiment was similar as in control cells (Figure 4E). To quantify the recovery process in more detail, we fitted the time-dependent fluorescence signal with exponential functions (Figure 4C). In the absence of ZC4H2, the recovery phase was generally well described using a monoexponential function, with an exponential time constant of 371 ± 83 s (Figure 4C,F). In contrast, two exponentials were required to describe the recovery of TRPV4-GFP fluorescence in cells expressing ZC4H2. The fast kinetic component was characterized by an exponential time constant of 23.7 ± 4.9 s, compared to 457 ± 71 s for the slow kinetic component (Figure 4C,F), the latter value being similar to the monoexponential time constant in the control cells. The estimated amplitudes of the fast kinetic component amounted to approximately 40% of the total recovery in the ZC4H2-expressing cells (Figure 4G). Note that we monitored the recovery phase for 800 s following bleaching, at which time point the recovery phase did not reach a steady state in a significant fraction of the cells. Therefore, a potential effect of ZC4H2 on slower components of the recovery process cannot be excluded at this point.

## 3. Discussion

In this study, we provide molecular and functional evidence for an interaction between the ion channel TRPV4 and the zinc-finger domain-containing protein ZC4H2. Based on an MAPPIT screen, ZC4H2 was identified as one of the strongest interactors with the cytosolic N terminus of TRPV4, and this physical interaction was confirmed using co-immunoprecipitation in a heterologous expression system. Using [Ca^2+^]_i_ imaging, we further provide evidence that ZC4H2 enhances basal TRPV4 activity and potentiates responses to chemical ligands and osmotic cell swelling. ZC4H2 did not have an effect in cells expressing the closest homologue TRPV3, excluding a general effect on TRP channel activity or on cellular Ca^2+^ handling. Using TIR-FRAP experiments, we further demonstrate that ZC4H2 has a pronounced effect on the perimembrane dynamics of TRPV4. Indeed, we found that ZC4H2 accelerates both the bleaching and the recovery from bleaching of TRPV4-GFP, indicative of a faster delivery to and retrieval from the plasma membrane. These findings may point towards a larger pool of rapidly recycling TRPV4-containing periplasmic vesicles in cells expressing ZC4H2, potentially affecting the number of functional TRPV4 channels at the plasma membrane. Our biochemical and imaging experiments seem to argue against a pronounced effect of ZC4H2 on the number of functional channels, as they did not reveal any significant difference in the steady-state levels of TRPV4-GFP at the plasma membrane. However, we acknowledge that these approaches may not be sensitive enough to pick up changes in protein levels in the range of 20%, which would nevertheless be sufficient to explain the observed effects on [Ca^2+^]_i_. Moreover, there is evidence that TRPV4-mediated responses to mechanical and chemical stimuli not only depend on the activation of channels that were already present in the membrane before onset of the stimuli but also on the rapid recruitment of additional channels from a pool of rapidly cycling perimembrane vesicles [27,28,29]. Finally, we cannot exclude at this point that the altered trafficking and increased functionality represent two distinct actions of ZC4H2 on the cellular behavior of TRPV4. Taken together, we conclude that ZC4H2 represents a newly identified regulator of TRPV4 that interacts with the N-terminus, and thereby regulates channel function and turnover at the plasma membrane.

In an earlier work, several other cytosolic proteins interacting with the N terminus of TRPV4 have been identified [2] (http://trpchannel.org/summaries/TRPV4), including Osteosarcoma Amplified 9 (Os-9), Protein Kinase C And Casein Kinase Substrate In Neurons 3 (Pacsin3), and atrophin-interacting protein 4 (AIP4), but their effects on TRPV4 differed from what we found for ZC4H2. Binding of Os-9 to the N terminus of TRPV4 lowers the levels of TRPV4 in the plasma membrane by inhibiting the release of the channel from the endoplasmic reticulum (ER) [30]. In contrast, the interaction of the SH3 domain of Pacsin3 with a proline-rich domain in the N terminus of TRPV4 increases the levels of the channel in the plasma membrane but inhibits basal activity as well as responses to heat and cell swelling [31,32]. Finally, the homologous to E6-AP carboxyl terminus (HECT)-family ubiquitin ligase AIP4 reduces plasma membrane levels of TRPV4 by facilitating its endocytosis [33,34]. Moreover, evidence has already been presented in an earlier work that channel-activating stimuli, such as shear stress or the agonist GSK 1016790A, can induce the recruitment of intracellular pools of TRPV4 to the plasma membrane [27,28,29]. It remains to be elucidated how these signals and interacting partners of TRPV4 contribute to functional channel expression at the plasma membrane in health and disease. Further research into the ZC4H2–TRPV4 interaction may provide further insights into TRPV4 channel trafficking and turnover at the membrane. 

At this point, the cellular function of ZC4H2 is not well understood, and, as for TRPV4, the link between specific mutations in the *ZC4H2* gene and the resulting disease symptoms is elusive [16,21,23]. ZC4H2 contains a C-terminal zinc-finger domain characterized by four cysteine and two histidine residues. Expression is predominately found in the brain and spinal cord in the embryonic stage [20]. ZC4H2 also contains a nuclear localization signal, and shuttles between the nucleus and the cytosol [20,35]. ZC4H2 has been shown to interact with Smad signaling proteins [23] and with the ubiquitin E3 ligase Rnf220 [22,36], and disease causing mutations may influence the transcriptional regulation of specific genes by these targets, thereby affecting neuronal development. Of note, although zinc-finger-containing proteins are best known in the context of transcription in the nucleus, it is not uncommon that they affect protein function via direct interaction [37]. Notably, many disease-causing mutations in the *ZC4H2* gene affect the nuclear localization signal, leading to a more abundant presence in the cytosol [35,38]. It will be of great interest to further investigate whether such cytosolic mutants are more prone to enhance TRPV4 function, and thereby provoke disease symptoms that are common to TRPV4-pathies and ZARDs. 

## 4. Materials and Methods

### 4.1. Cell Culture

Human embryonic kidney cells (HEK-293T) were grown in Dulbecco’s modified Eagles medium (DMEM) containing 10% (*v/v*) fetal calf serum, 2 mM L-glutamine, 2 U/mL penicillin, and 2 mg/mL streptomycin (Gibco/Invitrogen, Carlsbad, CA, USA) at 37 °C in a humidity-controlled incubator with 10% CO_2_. Regularly, potential cell contamination with mycoplasma species was tested using PlasmoTest—Mycoplasma Detection kit (InvivoGen, Toulouse, France). Cells were transiently transfected with 1 µg cDNA encoding either ZC4H2-mCherry or mCherry cloned in the pcDNA3.1 vector together with 1 µg of human TRPV4-GFP cloned in the PCIneo-vector, using TransIT^®^-293 Transfection Reagent (Mirus Corporation, Madison, WI, USA). On the next day, cells were reseeded on poly-l-lysine-coated (0.1 mg/mL) 25-mm glass coverslips with thickness of 0.16–0.19 mm (Gerhard Menzel GmbH, Braunschweig, Germany) for TIRF experiments, or 18-mm glass coverslips with thickness of 0.13–0.16 mm (Gerhard Menzel GmbH) for Ca^2+^ imaging.

### 4.2. Protein Expression Analysis 

The whole cell lysis buffer consisted of (in mM) 50 HEPES, 150 NaCl, 1.5 MgCl_2_, 1 EDTA, 1 PMSF, 10% glycerol, 1% Triton X-100, supplemented with protease inhibitor cocktail (Sigma-Aldrich, Bornem, Belgium). For immunoprecipitation, ChromoTek’s GFP-Trap^®^ coupled to magnetic agarose beads (ChromoTek, Planegg, Germany) was used according to the manufacturer’s protocol to pull down GFP-fused hTRPV4. For purification of the membrane protein fraction, biotinylation assays were performed on co-transfected HEK-293T cells using EZ-Link™ Sulfo-NHS-SS-Biotin (1 mg/mL for 30 min, Thermofisher Scientific, Massachusetts, MA USA) before whole cell lysis. Thereafter, biotin-bound membrane proteins were isolated using Pierce^TM^ Streptavidin Agarose Resin beads (Thermofisher Scientific, Massachusetts, MA, USA) according to the manufacturer’s protocol. Next, whole cell lysate, bead-bound fractions, wash fractions, and biotinylated fractions were prepared for SDS-page by adding 4-fold concentrated Laemmli sample buffer (Biorad, California, CA, USA) substituted with 2-β mercaptoethanol (99%, Sigma-Aldrich, Bornem, Belgium) and heating to 95° for 5 min. Samples were evaluated by SDS-PAGE using NuPAGE Novex Bis-Tris 4%–12% Gels (Life Technologies, Carlsbad, CA, USA) according to the manufacturer’s protocol. Separated proteins were transferred to a PVDF membrane (Millipore, Billerica, MA, USA) and immersed for 1 h in blocking solution (5% *w/v* nonfat dry milk in TBS containing 0.1% Tween-20). The membranes were probed with anti-hTRPV4 (in-house), ZC4H2 (ab100924, Abcam, 1/1000), β-actin (A1978, Sigma, 1/4000), and Na/K-ATPase (ab7671, Abcam, 1/2000) antibodies overnight at 4 °C. Next, the membranes were washed in TBST and incubated with horseradish peroxidase (HRP)-conjugated secondary antibodies (1/5000; Cell Signaling Technology Inc, Beverly, MA, USA) for 1 h at room temperature. Immunoreactive complexes were visualized using ECL Western blotting detection reagent (GE Healthcare, Buckinghamshire, UK) and ChemiDoc MP Imaging System (version 5.01 Beta, Bio-rad Laboratories, Hercules, CA, USA). Results were analyzed using Image Lab Software (version 5.01 Beta, Bio-Rad Laboratories). After visualization, membranes were stripped (Re-blot plus mild solution, Merck-Millipore, Massachusetts, MA, USA), washed, blocked, and reblotted using the above described procedure. Quantification of protein bands was performed using Fiji analysis software.

### 4.3. MAPPIT

The mammalian-based screening tool MAPPIT (MAmmalian Protein–Protein Interaction Trap) [15] was used to identify interactors of the TRPV4 protein. The screening was performed using the hORFeome v8.1 library [18] and ORFeome Collaboration clones [19] as preys in HEK-293T cells. The bait consisted of the full intracellular N-terminus of TRPV4 (AA 1–466).

### 4.4. qPCR

RNA extraction from samples was done using RNeasy kits (Qiagen, Hilden, Germany) according to the manufacturer’s instructions, followed by reverse transcription into cDNA using Ready-To-Go Youprime First-Strand beads (GE healthcare Life Sciences, Buckinghamshire, UK). Quantitative real-time PCR was carried out using specific TaqMan^TM^ gene expression assays accompanied with TaqMan universal mastermix (Applied Biosystems^®^, Foster City, CA, USA). Experiments were performed using a 7500 Real Time PCR system and software (Applied biosystems, Lennik, Belgium) using a 40-replication cycle protocol. Sample thresholds were analyzed relative to the housekeeping gene Hypoxanthine Phosphoribosyltransferase (HPRT) or GAPDH as control (ΔC_t_), and data are presented as (2^−ΔCt^). Statistical analysis was performed on the average ΔC_t_ from three technical replicates for each sample. Tissues used were dorsal root ganglia (DRG), embryonic spinal motor neuron tissue, and primary osteoclasts, chondrocytes, and osteoblasts.

### 4.5. Ca^2+^ Imaging

For intracellular Ca^2+^ measurements, cells were incubated for 30min with 2µM Fura-2 acetoxymethyl (AM) ester (Biotium, Hayward, CA, USA) in cell culture medium. Fluorescent signals were evoked during alternating illumination at 340 and 380 nm using an MT-10 illumination system (Tokyo, Japan) and CellM software from Olympus. Absolute Ca^2+^ concentrations were calculated from the ratio of these fluorescent signals as described before [39]. Experiments were performed at room temperature with standard extracellular perfusion buffer containing (in mM) 150 NaCl, 2 CaCl_2_, 1 MgCl_2_, 10 HEPES, adjusted to pH 7.4 using NaOH. For stimulation of the cells, the following modulators were used: GSK1016790A (10 nM), 4αPDD (10 µM), arachidonic acid (10 µM), and ionomycin (1 µM), all from Sigma-Aldrich (Bornem, Belgium). In experiments where osmotic responses were analyzed, we used a modified isotonic solution containing (in mM): 105 NaCl, 2 CaCl_2_, 1 MgCl_2_, 10 HEPES, and 90 mannitol, adjusted to pH 7.4 using NaOH. A hypotonic solution was obtained by omitting mannitol from the modified isotonic solution.

### 4.6. TIRF (Total Internal Reflection Fluorescence) Microscopy

For TIRF experiments, cells were maintained in extracellular solutions containing (in mM): 150 NaCl, 6 KCl, 2 CaCl_2_, 1,5 MgCl_2_, 10 HEPES, adjusted to pH 7.4 with NaOH. Images were obtained at room temperature through an inverted Zeiss Axio Observer.Z1 microscope with 100X oil objective (numerical aperture of 1.45) and a Hamamatsu Orca-R2 camera, using Axiovision software. Fluorescence excitation was performed with multidimensional acquisition using 488-nm (GFP) and 561-nm (mCherry) lasers at 2% of their maximal power. The incident fluorescent excitation angle in TIRF mode was adjusted based on the laser wavelength (between 65° and 68°) to evoke an evanescent wave with an expected decay length constant of 120 nm. Time series were recorded at intervals of 500 ms, and constant focus was guaranteed by use of Zeiss Definite Focus module. Microscopy images were analyzed using AxioVision 4.8 digital image processing software (Zeiss), ImageJ and Origin 7.0 (OriginLab Corporation, Northampton, MA, USA).

For the TIR fluorescence recovery after photo-bleaching (FRAP) experiments, bleaching of the samples was performed using full laser power (100%) at 400ms exposure for 5 frames with an interval of 500ms. The recovery phase was measured at 3% laser power, with an exposure of 50ms for 40 frames with an interval of 20 s.

### 4.7. Data Analysis

Data were analyzed using Igor Pro and WinASCD software. Statistical analysis (unpaired *t*-tests) and display was done using Origin 9.0. *p*-values < 0.05 were considered significant (*).

## Abbreviations

AA	arachidonic acid
ARD	ankyrin repeat domain
CMT	Charcot-Marie-Tooth
FDAB	familial digital arthropathy-brachydactyly
MAPPIT	Mammalian Protein-Protein Interaction Trap
SD	skeletal dysplasias
SMA	spinal muscular atrophy
TIRF	total internal reflection fluorescence
TIR-FRAP	Total Internal Reflection - Fluorescence Recovery After Photo-bleaching
TRP channel	Transient Receptor Potential channel
TRPV4	TRP channel Vanilloid 4
ZARDs	ZC4H2-associated rare disorders
